# Intergenerational Sex-Specific Transmission of Maternal Social Experience

**DOI:** 10.1038/s41598-018-28729-8

**Published:** 2018-07-12

**Authors:** Jamshid Faraji, Mitra Karimi, Nabiollah Soltanpour, Zahra Rouhzadeh, Shabnam Roudaki, S. Abedin Hosseini, S. Yaghoob Jafari, Ali-Akbar Abdollahi, Nasrin Soltanpour, Reza Moeeini, Gerlinde A. S. Metz

**Affiliations:** 1 University of Lethbridge, Canadian Centre for Behavioural Neuroscience, Lethbridge, Canada; 2 Golestan University of Medical Sciences, Faculty of Nursing & Midwifery, Gorgan, Iran; 3 Iran Ministry of Education-Exceptional Education Organization, Inclusive-Integrated Education Program for Children with Special Needs, Tehran, Iran; 4 Babol University of Medical Sciences, Department of Anatomical Sciences, Babol, Iran; 5 Islamic Azad University, Department of Psychology, Sari Branch, Sari, Iran; 6 Avicenna Institute of Neuroscience, Department of Behavioural Studies, Yazd, Iran

## Abstract

The social environment is a major determinant of individual stress response and lifetime health. The present study shows that (1) social enrichment has a significant impact on neuroplasticity and behaviour particularly in females; and (2) social enrichment in females can be transmitted to their unexposed female descendants. Two generations (F0 and F1) of male and female rats raised in standard and social housing conditions were examined for neurohormonal and molecular alterations along with changes in four behavioural modalities. In addition to higher cortical neuronal density and cortical thickness, social experience in mothers reduced hypothalamic-pituitary-adrenal (HPA) axis activity in F0 rats and their F1 non-social housing offspring. Only F0 social mothers and their F1 non-social daughters displayed improved novelty-seeking exploratory behaviour and reduced anxiety-related behaviour whereas their motor and cognitive performance remained unchanged. Also, cortical and mRNA measurements in the F1 generation were affected by social experience intergenerationally via the female lineage (mother-to-daughter). These findings indicate that social experience promotes cortical neuroplasticity, neurohormonal and behavioural outcomes, and these changes can be transmitted to the F1 non-social offspring in a sexually dimorphic manner. Thus, a socially stimulating environment may form new biobehavioural phenotypes not only in exposed individuals, but also in their intergenerationally programmed descendants.

## Introduction

The use of an enriched environment (EE)^1–3^ represents a classic and highly effective intervention in animal studies. While rodents in the standard housing condition are mostly raised in small groups of 2–3 in standard shoebox cages and are served with regular food chow and water, housing in an EE exposes them to larger, complex spaces and an opportunity for social interaction with usually between 5–8 conspecifics^4^. Rodents housed in an EE paradigm are often exposed to rich sensory stimulation through introduction of a variety of objects with different shapes, sizes, colours and textures. In addition, motor stimulation in the EE is provided by opportunities for voluntary physical activity on running wheels, balance platforms, and climbing apparatuses. A combination of these environmental interventions represent the key features of an effective EE that includes a range of opportunities for social, motor, visual, somatosensory, gustatory and olfactory stimulation^5^. While the combined physical and social interaction has characterized the design of most EE interventions^6,7^, an alternative hypothesis is that mere social stimulation suffices to change brain anatomy and behaviour of rats^8^. The latter hypothesis, however, has been previously disputed under the assumption that inanimate stimuli must be taken into account^1^.

A typical EE has been defined as a rich combination of complex inanimate and social stimulation^1^. In response to Welch and others^8^, Rosenzweig’s team reported^1^ that social grouping alone is not sufficient to explain the cerebral effects of EE interventions in rats. Surprisingly, for almost 40 years now, albeit with very few exceptions^9^, this notion has not been questioned nor systematically investigated. The model used in the present study extends the classic definition of EE^8^ by isolating a single element of the EE, social stimulation, from the other constituents (e.g. cognitive, sensory and motor stimulation).

Here, we proposed that social experiences are critical influences on offspring phenotype. The present study investigated the sex-specific impact of social enrichment on the parental F0 generation and their F1 offspring. Male and female F0 and F1 rats were raised in two housing conditions, standard and social environments, to reach adulthood. A novel corridor field task was developed to assess sex-specific novelty-seeking behaviours. The neuromorphological and behavioural findings revealed that the female F0 brain and behaviour are particularly susceptible to social experience. More importantly, F0 social mothers transmitted the new phenotype formed by social experiences to only their F1 female offspring. Thus, behavioural programming by social experiences transmitted to the next generation arguably depends on a lineage-dependent mechanism.

## Materials and Methods

### Animals

Eighty eight male and female Wistar rats (290–485 g), bred and raised at the local vivarium, were used in this study. Animals were housed at room temperature (21–24 °C) on a 12-hour light/dark cycle (lights on at 7:30) with *ad libitum* access to food and water. Body weight was recorded every three days. Prior to behavioural testing rats were handled for approximately 3–4 min daily for three consecutive days. All behavioural training and testing was performed during the light phase of the cycle at the same time of day by an experimenter blind to the experimental groups. All procedures in this study were carried out in accordance with the National Institute of Health Guide to the Care and Use of Laboratory Animals and were approved by the Avicenna Institute of Neuroscience (AINS) Animal Care Committee.

### Experimental Design

#### Experiment 1

F0 pups and their mothers were left undisturbed from postnatal day (PND) 1–21. After weaning at PND 21, 48 pups gathered from 5 different litters were randomly assigned to four experimental groups in two housing conditions: (1) standard housing (males, n = 12), (2) standard housing (females, n = 12), (3) social housing (males, n = 12), and (4) social housing (females, n = 12). After living in either standard or social-housing conditions for 96 days, all F0 animals were subjected to blood sampling (Day 97) and behavioural assessments (Days 98–101). Once behavioural assessments were completed on day 101, five rats from each group were maintained for breeding, and 7 rats were sacrificed for histological assessment. Pairing for mating was counterbalanced across groups; five males from standard housing condition and 5 females from social housing condition were paired. Conversely, 5 males from the social housing group were paired with 5 females from the standard housing group. After detection of a vaginal plug, the male rats were removed from the cage, and females were kept individually during pregnancy with free access to food and water until delivery. Some of their offspring was assigned to Experiment 2. Only litters consisting of 8–12 pups were used in order to control for the effect of litter size on early dam-offspring relationships.

#### Experiment 2

**Figure 1:**
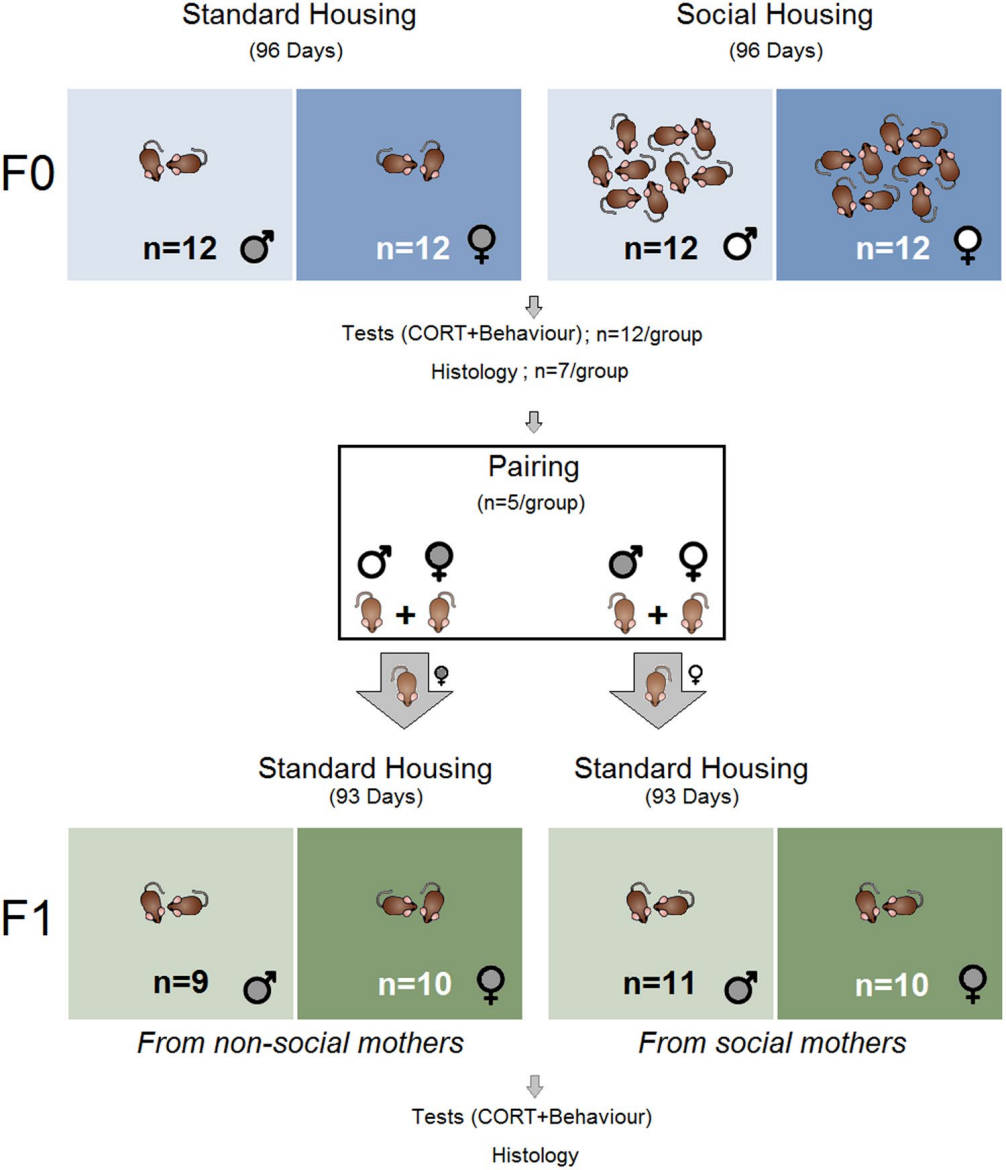
*Experimental design*. F0 and F1 generations (males and females) were exposed to either standard and social housing conditions. *Experiment 1*(*F0*)*:* Male and female pups gathered from 5 different litters were randomly assigned to four experimental groups (n = 12/group) in two housing conditions at postnatal day 21. While standard animals were kept for 96 days in standard housing in groups of 2–3, social animals were raised in groups of 12 for the same period of time. On days 97–101 behavioural assessments were completed. Five rats per group were maintained for pairing, and 7 rats were used for tissue collection. *Mating:* Five standard males and 5 social females, and also 5 social males and 5 standard females were paired for mating. *Experiment 2*(*F1*)*:* Forty pups from F0 dams who were previously raised in either standard or social housing conditions were randomly selected for Experiment 2 (F1). Based on their mothers’ housing conditions, the F1 offspring were then split into males and females (n = 9–11/group) and housed in groups of 2–3 for 93 days. All F1 animals were euthanized for histological assessments after behavioural testing was completed on days 94–98.

All pups grew up only with their mothers. At weaning, 19 pups (males, n = 9; females, n = 10) from F0 dams who were previously raised in the standard housing condition, and 21 pups (males, n = 11; females, n = 10) born to socially-housed dams were randomly selected for Experiment 2. Based on their mothers’ housing conditions, the F1 offspring (n = 40 in total) were then split into four groups: (1) maternal standard housing (males, n = 10), (2) maternal standard housing (females, n = 10), (3) maternal social housing (males, n = 10), and (4) maternal social housing (females, n = 10). These rats were housed in groups of 2–3 within standard housing units for 93 days. Animals were subjected to blood sampling on day 94 and behavioural assessments on days 95–98. All F1 rats were then euthanized for histological assessments when behavioural testing was completed on day 98. The experimental design is illustrated in Fig. 1.

### Housing Condition

The same size of polycarbonate cage was used for the social and standard housing conditions. F0 animals assigned to the social housing condition were housed and raised in groups of twelve within social housing units (86 cm × 86 cm × 41 cm). Because the present study was designed to focus on the impact of social interaction, no additional enrichment stimuli, such as objects, toys or additional nesting materials, were provided in the social housing condition. Also, food location and type were not changed. Animals were constantly living in the same units until completion of the experiment. Rats assigned to standard housing conditions were housed and raised in non-sibling groups of 2 or 3 in standard size cages (86 cm × 86 cm × 41 cm). Experiment 1 varies the number of cage mates (standard, n = 2–3 vs. social, n = 12) while keeping the size of the environments constant. In Experiment 2, however, all F1 animals (n = 9–10 per group) were housed and raised in groups of 2–3 within standard housing units (86 cm × 86 cm × 41 cm). Aspen wood mixed with shredded paper bedding material was used in both standard and social environments, and changed once per week. Animals were removed from their environments when bedding material was changed.

### Blood Samples and Corticosterone Assays

The procedure for blood sampling was previously reported by^10^. Blood samples (0.5–0.7 mL) were taken one day prior to behavioural assessments. Briefly, rats were placed in a restraint tube and blood samples were obtained by a tail notch with a scalpel blade. Blood samples were collected within the first 1–2 min in the tube to ensure circulating corticosterone (CORT) levels did not increase in response to the brief stress of the procedure. Blood samples were collected in heparinized tubes. Plasma was obtained by centrifugation at 8,000 rpm for 7 min. The plasma samples were stored at −20 °C until analyzed for CORT concentration using commercial radioimmunoassay kits (Salimetrics, UK). All samples were collected in the morning hours between 9:00 and 11:00 h. No behavioural testing was performed on blood sampling day. Three animals from Experiment 1 and one animal from Experiment 2 were excluded from CORT analysis due to some technical issues and/or insufficient blood samples.

### Behavioural Assessment

#### Novelty-Seeking Exploratory Behaviour

**Corridor Field Task (CFT)**: The CFT has been developed for the assessment of novelty-seeking exploratory behaviour in Experiment 1 (n = 42) and Experiment 2 (n = 39). The task consisted of a corridor field made of black Plexiglas measuring 165 cm by 165 cm by 35 cm in height. A segregated internal wall (30 cm in height) was placed in parallel with the external wall making a corridor (15 cm width) around the arena where the animals were able to freely move. Also, four small passages inserted on the internal wall of the apparatus allowed animals to expand their search outside the corridor and to the center of the task. The floor of the task was divided into three zones: (1) *corridor zone* comprising the zone between external and internal walls, (2) *open zone* comprising the area within the task excluding corridor and the central zones; and (3) *central zone* comprising the middle area of the arena (15 cm by 15 cm).

To assess different locomotor aspects of free exploration, two variations of the CFT were used: (1) plain CFT without a central object, and (2) CFT with a central object. All rats were individually allowed to freely explore the environment starting from one corner of the CFT arena facing the apparatus external wall. The same corner was used for all rats during a test period. The experimenter left the room immediately after placing the rat in the task. All test sessions lasted 8 min, during which performance was recorded under dim illumination by a ceiling-mounted camera (CCTV Auto tracking PTZ; SONY, Tokyo, Japan) and analysed by a computer tracking system (SINA motiongraph, V.II, 2011, Tabriz, Iran) through analysis of the time spent in each zone, and path speed. After each animal, the apparatus was cleaned with 70% alcohol.

#### Spatial Learning and Memory

**Morris Water Task (MWT)**: The hidden platform version of the MWT (151 cm diameter) was used to assess spatial performance, learning and memory^11^. Animals (Experiment 1: n = 28; Experiment 2: n = 28) were tested by a one-day testing protocol (10 trials per animal). The maximum duration of each swim trial was 60 s, and the location of the hidden platform (quadrant 3) remained constant from trial to trial. Movements of the animals including time spent to find the hidden platform (latency), swim length, swim speed and thigmotaxis (wall-hugging behaviour) were recorded and analyzed by an image-computerized tracking system (HVS Image 2020, UK). A no-platform probe trial (30 seconds) was also performed three hours after the completion of the single-session hidden platform testing as a measurement of reference memory. The percentage of time that the animals spent in each quadrant of the task was recorded.

#### Sensorimotor Balance

**Balance Beam Task (BBT)**: In the present study, the BBT was employed to test sensorimotor integration (coordination and balance; from^12^ with modifications). Animals of both experiments (Experiment 1: n = 40; Experiment 2: n = 32) were positioned on one end of a wooden round bar (2 cm wide, 125 cm long, 50 cm high) and their home cage was located at the other end of the bar. A foam pad was placed underneath to cushion falling animals. The animals were tested for three trials on the bar, and the latency to traverse the bar and number of times the hind feet slipped off the bar were recorded.

#### Anxiety-Related Behaviour

**Elevated Plus Maze (EPM)**: Anxiety-related behaviour was assessed in the EPM^13^ under dim illumination. The apparatus made of black Plexiglas consisted of two open and two closed arms (all 55 × 11 cm) and was elevated 83 cm above the floor. The open arms had no side or end walls, but the closed arms had side and end walls (35 cm high). Rats in Experiment 1 (n = 32) and 2 (n = 30) were placed individually in the central square facing either the left or right open arm, and were allowed to explore the maze for 5 min. The experimenter left the room immediately after placing the rat on the maze, and the behaviour of the animals in the maze was transmitted by a camera (CCTV Auto tracking PTZ; SONY, Tokyo, Japan) and analysed by a computer tracking system (SINA Motiongraph, V.II, 2011, Tabriz, Iran) via analysis of the standard measures (time spent in open and closed arms) of the EPM. Path speed and path length (distance traveled) in the maze were also analysed. In order to minimize olfactory cues, the apparatus was cleaned with 70% alcohol after testing each animal.

### Histological Assessment

Animals in Experiment 1 (F0 rats; n = 7/group) were euthanized and intracardially perfused^11^. Brains including olfactory bulb and cerebellum were removed and weighed. Brains were then fixed for coronal sectioning (40 µm) and cresyl violet staining. From the coronal sections, measurements included neuronal density (Gray Value Index; GVI) and cortical thickness in both hemispheres.

#### Neuronal Density and Cytoarchitectonics

Neuronal density analysis (quantitative cytoarchitectonics) was performed using ImageJ 1.47b (http://imagej.nih.gov/ij; NIH, USA). Three regions of interest (ROI) were determined between primary and secondary motor cortices (M1 and M2) and the secondary somatosensory cortical region of each hemisphere. Both left and right ROIs included the same cortical regions and mainly all six cortical layers. An absolute GVI within the ROIs was separately measured for each region^14^.

#### Cortical Thickness

Four points (medial, central, lateral, and ventrolateral) from each brain were selected^14^ based on coordinates by Paxinos and Watson^15^. The most rostral section measured was located at ~3.20 mm anterior to bregma and the most caudal section at ~−6.30 mm posterior to bregma. For each point, a vector was considered from the tangent of the outer edge to the inner edge of the cortex. ImageJ software 1.47b (http://imagej.nih.gov/ij; NIH, USA) was used to record up to eight measurements of cortical thickness from each coronal section, four from each hemisphere. Cortical thickness in the present experiment was a mean measure of both hemispheres in three consecutive slices.

#### Golgi-Cox Staining

Animals in Experiment 2 (F1 rats; n = 5–6/group) were also transcardially perfused after all behavioural testing was completed. All brains were extracted from the skull and weighed. Brains were then preserved in Golgi–Cox solution (30 ml) for 14 days in a dark location, and were transferred to 30% sucrose solution for 15 days^16,17^ with modification. Sectioning was performed on a vibratome at 200 µm, and the slices were mounted on gelatin-coated slides. All slices were stained according to published procedure^16^. The procedure allowed staining of entire dendrites of individual neurons. For examination, dendrites had to be completely stained and visible, free of stain precipitation and not affected by blood vessels. Neurons in the ROIs were drawn with a camera lucida. Prefrontal individual pyramidal cells (layers II-III) from the Golgi–Cox stained slices were selected and traced for analysis using a camera lucida mounted on a microscope at 200 × magnification. Neuromorphological measurements included apical and basilar Sholl analysis of dendritic length that intersect concentric circles spaced 20 μm apart on the concentric ring procedure of Sholl, dendritic branch and spine density or the number of spine protrusions on a 50-μm segment of dendrite traced at 1,000 × magnification^16,17^. In the present experiment, a total of 12 neurons (6 per hemisphere) were traced in each animal.

#### *In Situ* Hybridization

Animals (F1 rats; n = 4–5/group) were euthanized with an overdose of sodium pentobarbital at the end of Experiment 2 to rapidly remove the brains. Brains were frozen on dry ice and stored at −80 °C until further processed for *in situ* hybridization. The prefrontal cortex (PFC) was isolated from 1 mm thick frozen coronal sections using a blunted 16-gauge stainless steel needle based on the coordinates (between ~4.70–2.70 mm from bregma) for adult rat brains. All *in situ* hybridization procedures were carried out as previously described in detail^18^ with modifications) and all sections were run under identical experimental conditions. Briefly, brains were sectioned on a cryostat (15 µm). The fixed, air-dried sections were incubated overnight with two 33P-labelled 48-mer oligonucleotide probes in hybridization buffer and the excess and unbound probe was washed off. Brain sections were exposed to BioMax MR-1 X-ray films for 4 weeks. Levels of brain-derived neurotrophic factor (BDNF) mRNA were analyzed and masked by optical densitometry of autoradiographic films using a computerized image analysis system (MCID, Canada) and ImageJ 1.47b (http://imagej.nih.gov/ij; NIH, USA).

#### BDNF Protein Analysis

The enzyme-linked immunosorbent assay (ELISA) was used to quantify BDNF protein levels in the PFC^18^. Briefly, the tissues were homogenized in 100x (w/v) ice-cold homogenization buffer containing a protease-inhibitor cocktail and further diluted 1:9 in this buffer to a total dilution of 1000x^18^. ELISA was performed on the homogenate using the BDNF Emax Immuno Assay Systems (Promega KK, Tokyo, Japan).

### Statistical analysis

In all statistical analyses (SPSS 16.0, SPSS Inc., USA), a p-value of less than 0.05 was chosen as the significance level. Effects of main factors (Group; 4 levels, Sex; 2 levels, Generation; 2 levels, Litter; 5 levels) were analyzed for body weight (31–32 days), brain weight, plasma CORT, behavioural measures (time spent in each zone in CFT, latency, swim length and speed, thigmotaxis, and probe function in the MWT, time to cross the beam and foot slips on the BBT, and time spent in open and closed arms accompanied by path length and speed on the EPM) and neuromorphological indices (GVI, cortical thickness, dendritic complexity) along with molecular alterations (BDNF mRNA and proteins) by *repeated measures*, *one*- and *two*-*way* ANOVAs. Also a two-level Group effect (F0: standard vs. social or F1: standard [from non-social mothers] vs. standard [from social mothers]) was analysed where no significant difference between males and females was observed. *Post*-*hoc* Tukey test was used to adjust for multiple comparisons where needed. Familywise error was considered prior to the multiple *post*-*hoc* analyses if necessary. All data are presented as mean ± standard error.

## Results

### Social experience reduced HPA axis activity

**Figure 2:**
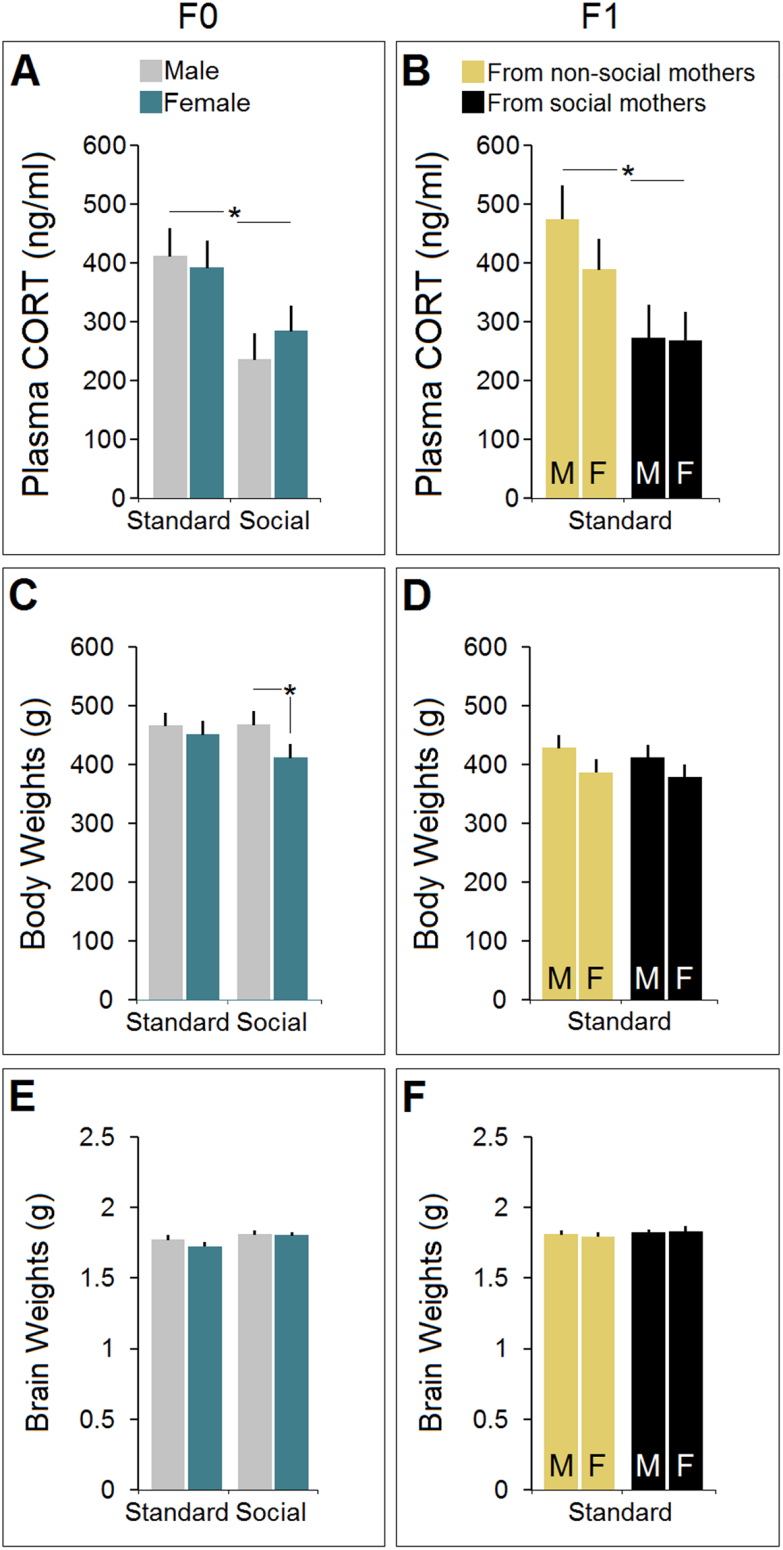
(**A**,**B**) *Changes in circulating CORT in F0 and F1 generations*. Social experience for 96 days significantly reduced plasma CORT levels in F0 rats (n = 12/group). F1 offspring that did not experience social interaction during postnatal development inherited the reduced level of HPA axis activity from their social mother. No significant difference was found between F0 social males and females. (**C**,**D**) *Effects of social experience on body weight in F0 and F1 generations*. Only F0 social females (n = 12) showed lower body weight when compared with other groups. (**E**&**F**) *Brain weight and housing conditions in F0 and F1 generations*. Despite a slight increase in brain weight, the F0 socially-housed animals were not significantly different when compared with standard rats. Asterisks indicate significant differences: *p ≤ 0.05; *One*-*Way* ANOVA. Error bars show ± SEM.

A summary of plasma CORT levels for both generations (n = 10–12/group) is shown in Fig. 2A,B. There was a significant effect of Group (standard-two levels vs. social-two levels; F_3,43_ = 14.11, p ≤ 0.02; *One*-*Way* ANOVA) linked to reduced CORT levels in social groups (399.59 ± 53 ng/ml vs. 259.28 ± 54 ng/ml) indicating that both male and female F0 animals raised in the social housing condition had lowered HPA axis activity, thus reduced level of circulating CORT (all p ≤ 0.05, *Post*-*hoc*; Fig. 2A). *One*-*Way* ANOVA did not reveal significant differences between litters (p ≥ 0.76).

Despite of being reared in standard housing conditions, F1 animals born to socially-housed mothers showed diminished levels of circulating CORT when compared with animals of the same generation whose mothers were raised in the standard housing condition (269.22 ± 51 ng/ml vs. 429.67 ± 48 ng/ml). A significant effect of Group (standard-two levels vs. standard-two levels; F_3,37_ = 13.84, p ≤ 0.01; *One*-*way* ANOVA) was found suggesting that HPA axis activity in F1 animals born to F0 social mothers was significantly impacted by the ancestral experiences (all p ≥ 0.05; *Post*-*hoc*; Fig. 2B). There was no significant difference between generations (p ≥ 0.37) or litters (p ≥ 0.59).

### Social experience reduced body weight only in F0 females

There was a significant main effect of Group (F_3,46_ = 6.37, p ≤ 0.04; *One*-*way* ANOVA) and Sex (F_1,46_ = 7.09, p ≤ 0.04; *One*-*way* ANOVA) in the F0 animals (n = 12/group) where only females in the social housing group showed lower body weight compared with other groups (411.5 g vs. 467.41 g, 451.5 g and 465.83 g; all p ≤ 0.05, *Post*-*hoc*, Fig. 2C). Furthermore, a significant effect of Litter (F_5,44_ = 13.11, p ≤ 0.046; *One*-*way* ANOVA, Table 1) was found through which litter 2 displayed lower body weight than other litters (all p ≤ 0.05, *Post*-*hoc*). Also, no effect of Group (p ≥ 0.84), Litter (p ≥ 0.89) and Sex (p ≥ 0.61) was found in the F1 rats in terms of body weight. Inter-generational comparisons did not show differences between F0 and F1 animals (p ≥ 0.08), in spite of slightly reduced body weight in the F1 generation (Fig. 2D).

**Table 1:** Litter effects in CORT response, body weight, and brain weight.

Litter	*N*=	CORT (ng/ml)	Body Weight (g)	Brain Weight (g)
F0				
L1	10	339 ± 51	457 ± 22	1.76 ± 0.03
L2	9	403 ± 38	396 ± 20*	1.73 ± 0.03
L3	10	362 ± 50	469 ± 22	1.74 ± 0.04
L4	10	399 ± 52	471 ± 21	1.72 ± 0.03
L5	9	344 ± 51	448 ± 21	1.74 ± 0.03
		*p* = *0*.*76*	*p < *0*.*05*	*p* = *0*.*16*
F1				
L1	7	409 ± 66	436 ± 24	1.63 ± 0.05
L2	7	338 ± 51	448 ± 23	1.70 ± 0.04
L3	9	390 ± 53	401 ± 20	1.68 ± 0.04
L4	8	372 ± 88	469 ± 26	1.72 ± 0.05
L5	9	286 ± 71	359 ± 21	1.71 ± 0.03
		*p* = *0*.*59*	*p* = *0*.*89*	*p* = *0*.*94*

### Social experience had no effect on brain weight

Despite a slight increase in brain weight, the F0 socially-housed animals were not significantly different from standard rats (1.82 ± 0.03 vs. 1.76 ± 0.03 g; p ≤ 0.73). Neither effect of Group (p ≥ 0.66) nor Sex (p ≥ 0.081), Litter (p ≥ 0.94) or Generation (p ≥ 0.94; Fig. 2E,F) was significant in F1 rats.

### Social experience generated sexually dimorphic novelty-seeking behaviours in F0 and F1 generations

**Figure 3:**
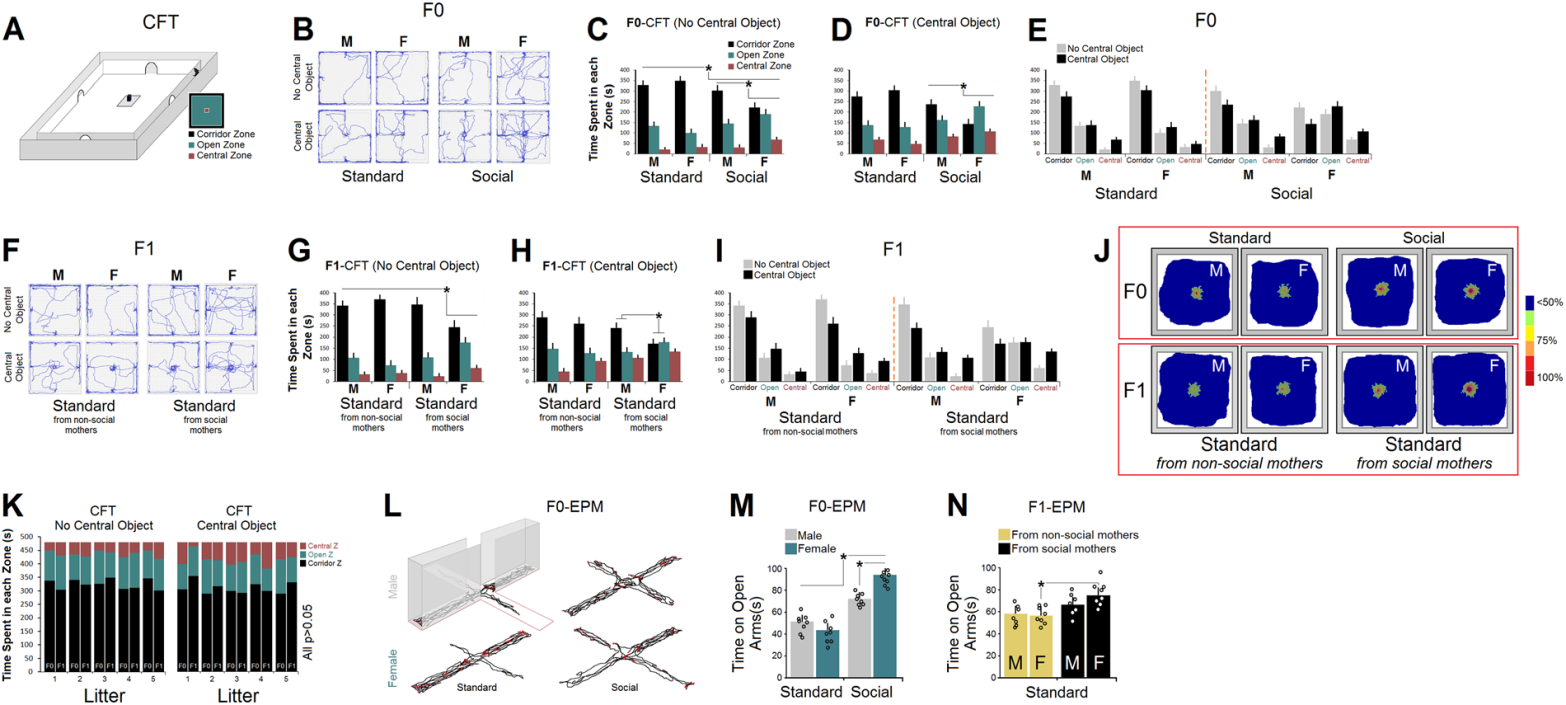
*Behavioural consequences of social experience in F0 and F1 generations*. (**A**) *Illustration of the corridor field task* (*CFT*). To assess novelty-seeking behaviour, rats were individually allowed to freely explore the environment (165 × 165 × 35 cm) for 8 minutes. Two variations were used: CFT without the central object, and CFT with a central object. (**B**) *Representative search paths of the F0 generation in the different CFT variations*. F0 social animals, particularly females, explored the open and central zones more than their standard housing counterparts. (**C**–**E**) *Time* (*seconds*) *spent in each zone of the CFT* (*corridor*, *open*, *central*) *by F0 generation*. Note that social animals spent significantly more time in the open and central zones in the no-central-object task variation. Social females spent less time in the corridor zone and explored the open and central zones more than their social male siblings (n = 11–12/group). Exploration in the central-object variation also showed that F0 social females spent significantly less time in the corridor zone, and more time in the open and central zones when compared with social males and standard groups. (**F**) *Representative path trajectories of the F1 generation in both CFT variations*. Only standard females born to social mothers spent more time exploring the open and central zones. (**G**–**I**) *Time* (*seconds*) *spent in each zone of the F1 generation in CFT*. Only standard female offspring born to social mothers spent less time in the corridor and more time in open and central zones than other groups. Like their social mothers, F1 female offspring spent significantly less time in the corridor and more time searching the open zones in the central-object CFT version (n = 9–11/group). (**J**) *Occupancy plots of paths during exploration of the central zone as an indicator for novelty*-*seeking behaviour*. Each plot compiled from individual tracks represents the percent time spent in the central zone (n = 6–8/group). (**K**) *Insignificant effect of Litter in F0 and F1 rats in terms of the time spent in different zones of the CFT*. Litters in the F0 and F1 generations indicated a similar pattern of search preference in different zones of the CFT with and without a central object. (**L**) *Representative path trajectories in the F0 generation in the elevated plus maze*. Social housing rats explored the open arms more than the standard housing rats. (**M**,**N**) *Time* (*seconds*) *spent in open arms for F0 and F1 generations*. Social F0 animals (n = 8/group) spent more time in open arms than standard animals. Also, standard housing female F1 offspring born to social mothers explored open arms significantly more than their standard female counterparts born to standard mothers (n = 7–8/group). Circles in the bar graphs represent individual animals. Asterisks indicate significant differences: *p ≤ 0.05; *One*-*way* and *Repeated*-*measures* ANOVA. Error bars show ± SEM.

An illustration of the corridor field task (CFT) along with paths taken by F0 rats are shown in Fig. 3A,B. *No central object*: Fig. 3C–E shows the exploratory behaviour of F0 rats (n = 11–12/group) in the CFT. There was a significant difference between Groups (standard-two levels vs. social-two levels, F_3,42_ = 7.96, p ≤ 0.04; *Repeated*-*measures* ANOVA) in the task with no central object in terms of the time spent in each zone. A significant main effect of Sex (F_1,42_ = 16.28, p ≤ 0.02; *Repeated*-*measures* ANOVA) and Zone (F_2,42_ = 11.64, p ≤ 0.03; *Repeated*-*measures* ANOVA), but not Litter (p ≥ 0.43) was also observed. A *two*-*way* ANOVA did not show interactions between factors, except for Sex × Litter (p ≤ 0.04) and Litter × Generation (p ≤ 0.04). Post-hoc analysis also indicated a significant difference between F0 social males and females in all zones (all p ≤ 0.05; *Post*-*hoc*, Fig. 3C) suggesting that social females spent less time in the corridor and more time in the open and central zones of the CFT when compared with standard housed animals and their male counterparts. *Central object*: A significant effect of Group (F_3,42_ = 11.47, p ≤ 0.03) and Sex (F_1,42_ = 23.59, p ≤ 0.02; *Repeated*-*measures* ANOVA) was found in the CFT with the central object. No significant effect of Litter was observed (p ≥ 0.07). Also, a one-way ANOVA revealed a significant difference between social males and females in terms of the time spent in each zone (Corridor: 304 ± 23 s vs. 222 ± 20 s, F_1,22_ = 18.04, p ≤ 0.02; Open: 145 ± 19 s vs. 190 ± 17 s, F_1,22_ = 13.81, p ≤ 0.03; Central: 31 ± 6 s vs. 68 ± 4 s, F_1,22_ = 9.36, p ≤ 0.03; Fig. 3D). *Path speed*: Examination of path speed during exploration in both CFT tests showed that F0 social females had a slightly faster exploration in the fields than social males and standard rats (No central object: [males] 0.117 m/s vs. [females] 0.121 m/s; Central object: [males] 0.122 m/s vs. [females] 0.126 m/s). There was, however, no significant difference between groups in terms of path speed (data not shown).

F1 rats (n = 9–11/group) displayed a similar profile of novelty-seeking behaviour in the CFT as did the F0 generation. *No central object*: Samples of path trajectories taken by F1 animals are shown in Fig. 3F. Significant effects of Group (F_3,34_ = 8.51, p ≤ 0.04; *Repeated*-*measures* ANOVA) and Sex (F_1,34_ = 13.09, p ≤ 0.04; *Repeated*-*measures* ANOVA) as well as an effect of Zone (F_2,34_ = 33.59, p ≤ 0.001; *Repeated*-*measures* ANOVA) but not Litter were found. The interactions between factors were not significant (all p ≥ 0.05; *Two*-*way* ANOVA), except for Sex × Litter (p ≤ 0.03) and Sex × Litter × Generation (p ≤ 0.05). A one-way ANOVA also indicated a significant difference between males and females whose mothers were raised in social housing conditions (Corridor: 347 ± 26 s vs. 243 ± 23 s, F_1,19_ = 29.66, p ≤ 0.02, Open: 108 ± 18 s vs. 176 ± 21 s, F_1,19_ = 19.01, p ≤ 0.03; Central: 25 ± 4 s vs. 61 ± 3 s, F_1,19_ = 10.47, p ≤ 0.03; Fig. 3G) suggesting that daughters of socially-reared mothers explored the arena more than their male siblings. Moreover, when compared with females whose fathers were raised in the social housing condition, female rats from socially-housed mothers explored more open and central zones of the CFT (Corridor: 369 ± 24 s vs. 243 ± 23 s, F_1,19_ = 26.14, p ≤ 0.03, Open: 72 ± 19 s vs. 176 ± 21 s, F_1,19_ = 13.86, p ≤ 0.02; Central: 39 ± 5 s vs. 61 ± 3 s, F_1,19_ = 11.52, p ≤ 0.04; *One*-*way* ANOVA). Thus, socially-housed fathers failed to transfer the new behavioural phenotype to their daughters. No significant difference was found between male and female rats whose fathers were raised in the social housing condition in terms of time spent in each zone (all p ≥ 0.05). *Central object*: A significant main effect of Group (F_3,34_ = 19.33, p ≤ 0.03; *Repeated*-*measures* ANOVA) and Sex (F_1,34_ = 14.63, p ≤ 0.02; *Repeated*-*measures* ANOVA) as well as Zones (F_2,34_ = 27.43, p ≤ 0.01; *Repeated*-*measures* ANOVA) but not Litter was found with a central object in the CFT. ANOVA showed that females whose mothers were raised in a social environment spent significantly less time in the corridor (241 ± 23 s vs. 169 ± 23 s, F_1,19_ = 21.39, p ≤ 0.03; *One*-*way* ANOVA), and more time in the open zone (133 ± 21 s vs. 177 ± 25 s, F_1,19_ = 11.46, p ≤ 0.04; *One*-*way* ANOVA) than their male siblings in spite of being raised in the standard housing condition. No difference was found between groups in terms of the time spent in the central zone (106 ± 14 s vs. 134 ± 13 s, p ≥ 0.33; Fig. 3H,I). A comparison between female groups whose fathers or mothers were socially-housed showed that the latter spent significantly less time in the corridor (261 ± 25 s vs. 169 ± 23 s, F_1,19_ = 42.76, p ≤ 0.001; *One*-*way* ANOVA), and more time in the open (127 ± 25 s vs. 177 ± 25 s, F_1,19_ = 13.06, p ≤ 0.04; *One*-*way* ANOVA) and central (92 ± 13 s vs. 134 ± 13 s, F_1,19_ = 14.27, p ≤ 0.03; *One*-*way* ANOVA) zones. *Path Speed*: No significant effect of Group and Sex (both p ≥ 0.05) was found in the F1 generation in terms of path speed within the CFT fields (No central object: [males] 0.121 m/s vs. [females] 0.119 m/s; Central object: [males] 0.126 m/s vs. [females] 0.124 m/s; data not shown). In summary, social experience alone appears to affect the novelty-seeking exploratory behaviour in the CFT in a sexually dimorphic manner. Occupancy plots of paths taken by rats (averaged for 6–8 animals/group) during 8-min exploration in the central zone of the CFT as an indicator for novelty-seeking behaviour is shown in Fig. 3J. Also, Fig. 3K profiles an insignificant Litter effect in F0 and F1 rats in terms of the time spent in different zones of the CFT (all p ≥ 0.05).

### Spatial navigation was not affected by social experience

Both generations have shown comparable profiles of spatial navigation in the MWT. Latency measurements over 10 trials of testing decreased across groups suggesting that all rats, regardless of their generations and sex were able to acquire and retrieve the spatial information in a similar rate. Repeated-measures ANOVA showed a significant effect of Trial (F0: F_9,25_ = 11.37, p ≤ 0.04; F1: F_9,25_ = 17.86, p ≤ 0.03) but no effect of Group (all p ≥ 0.05). Group by Trial effect in both generations was also significant (all p ≥ 0.05). No difference was found in swim length and speed among groups and generations (all p ≥ 0.05). The percentage of time that an animal spent swimming within 15 cm of the wall of the MWT (thigmotaxis) during training was also analysed to gain insights into anxiety-like behaviours. Thigmotaxis decreased in all groups as testing proceeded. However, no significant effect of Group was observed (p ≥ 0.05), despite a slight decrease of thigmotaxis in F0 social animals. Also, F1 males and females spent a similar proportion of time engaged in thigmotaxis and no difference was found between groups (p ≥ 0.05). *Probe trial*: Rats in both generations showed similar preference to spend time in the target quadrant (quadrant 3) within the MWT. No significant group difference was found in the time spent in the target quadrant (data not shown).

### Social experience had no effect on sensorimotor integration

The BBT was used to test motor coordination and balance. The F0 social groups in Experiment 1 crossed the beam faster than F0 standard groups, however, this trend did not achieve significance (all p ≥ 0.05). No group or sex differences were found in the number of foot slips (all p ≥ 0.05; data not shown).

### Social experience reduced anxiety-related behaviours

Rats were tested in the elevated plus maze (EPM; Fig. 3L) to assess possible anxiolytic effects of housing conditions. Reduced levels of anxiety-related behaviours were found in F0 social females compared to social males as indicated by increased exploration of the open arms in F0 social females (94.22 ± 6.14 s [31.40%] vs. 72.11 ± 7.02 s [24.03%]; F_1,14_ = 19.66, p ≤ 0.02; *One*-*way* ANOVA). No difference was found between F0 standard males and females, however (51.47 ± 6.58 s [17.15%] vs. 43.68 ± 6.26 s [14.56%]; p ≤ 0.071). *One*-*way* ANOVA also showed a significant difference between F0 standard and social rats in terms of time spent in open arms (47.57 ± 6.42 s [15.85%] vs. 83.18 ± 6.58 s [27.72%]; F_3,28_ = 28.14, p ≤ 0.03; Fig. 3M) indicating that both social males and females spent more time in open arms when compared with standard animals. No group difference was found in terms of locomotor activity (path length and speed; all p ≥ 0.05). Analysis of exploration by F1 rats showed that only standard females born to social mothers explored open arms significantly more than their female counterparts born to standard housing mothers (74.91 ± 7.52 s [24.97%] vs. 56.27 ± 6.39 s [18.75%]; F_1,11_ = 16.04, p ≤ 0.04; *One*-*way* ANOVA, Fig. 3N). Furthermore, path length and speed in the EPM were not affected by housing condition and/or parental experience in F1 rats (all p ≥ 0.05).

### Social experience in the F0 generation increased neuronal density and cortical thickness

**Figure 4:**
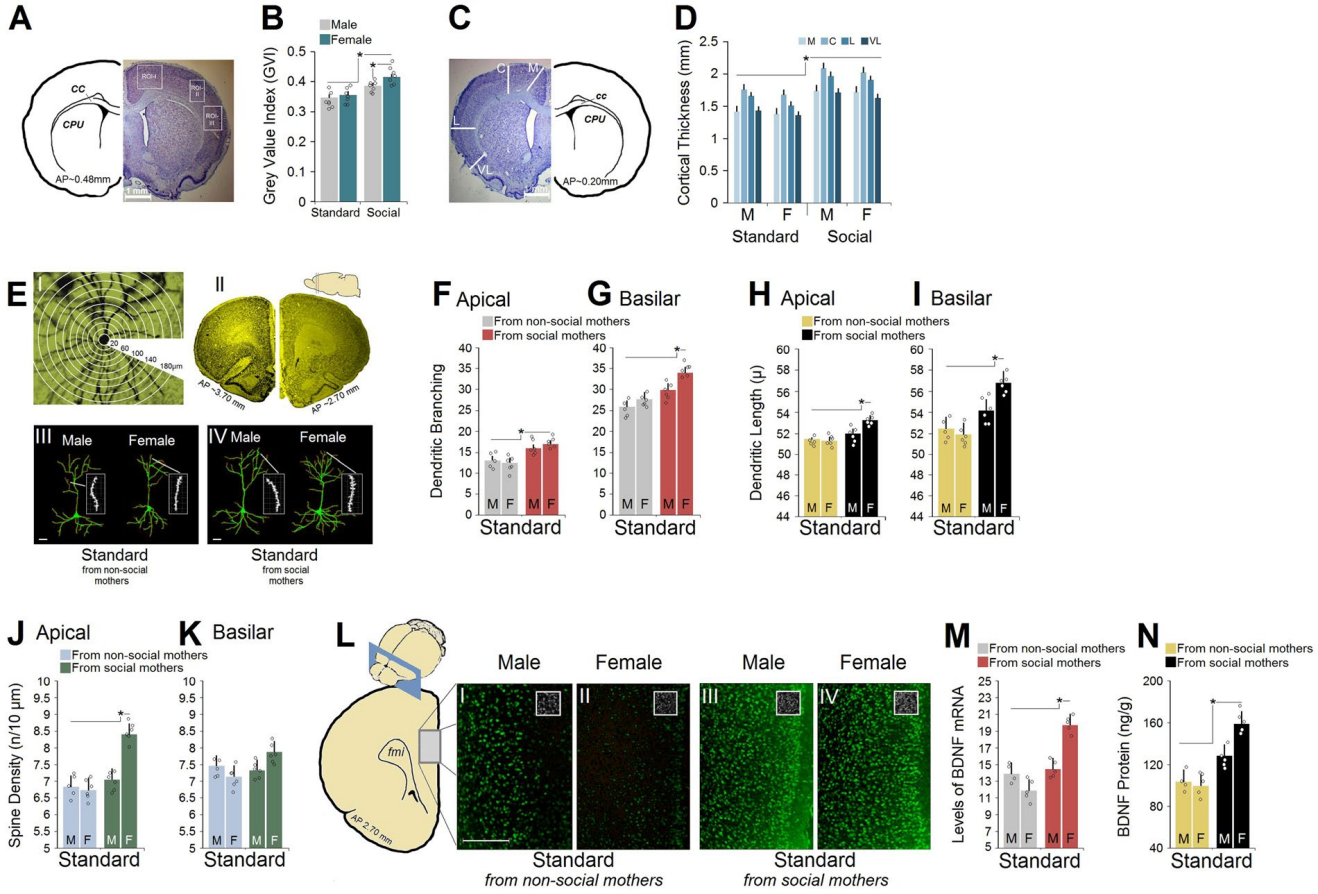
*Neuromorphological alterations by social experience in F0 and F1 generations*. (**A**,**B**) *Quantitative cytoarchitectonics in F0 generation*. Densitometry based on absolute gray value index (GVI) shown by approximate plane (plane 16, ~0.48 mm anterior to bregma) of stained brain sections. White squares represent three regions of interest (M2&M1, S1DZ, S1ULp) in cortical regions. Panel B: F0 social animals, particularly females, displayed an increased GVI (n = 6–7/group). (**C**,**D**) *Cortical thickness in the F0 generation*. Four points (medial, central, lateral, and ventrolateral) on three coronal brain sections were selected. Panel C shows a stained section and an atlas plate (plane 17, ~−0.20 mm posterior to bregma) from the right hemisphere. Panel D indicates increased thickness in all four cortical regions in F0 social animals (n = 6–7/group). (**E**) *i*: *Photomicrograph of a Golgi*-*Cox stained neuron as seen through the camera lucida*. Sholl analysis was employed for morphological changes including the dendritic length, dendritic branching and spine density. *ii*: *Representative Golgi*-*Cox stained coronal sections* (*AP* ~*3*.*70* *mm and AP* ~*2*.*70* *mm*; *200* *µm thickness*). A total of 12 prefrontal pyramidal neurons (6 per hemisphere) were traced per animal at 200× magnification. *iii & iv*: *Computer*-*assisted reconstructions of Golgi*-*Cox stained neurons* (*layers II*-*III*) *of mPFC*. (sub-panels, right) High-power representation of dendritic segments with dendritic spines (magnification 1000×) demonstrating increased number of spines in F1 non-social females born to F0 social mothers (IV-right) compared to other groups (n = 5–6/group; Scale bar = 20 μm). (**F**,**G**) *Dendritic branching in F1 generation*. Apical and basilar measurements indicated that F1 standard males and females born to social mothers had more dendritic branching than their standard housing counterparts (n = 5–6/group). Daughters of social mothers displayed more basilar branching than their male siblings. (**H**,**I**) *Dendritic length in F1 generation*. Only in females was apical dendritic length influenced by their mothers’ social experience. Basilar dendritic length was significantly affected in both sexes, particularly females, born to social mothers (n = 5–6/group). (**J**,**K**) *Spine density in F1 generation*. Apical spine density was highest in non-social F1 females born to social mothers. Basilar spine density was not affected by maternal social experience (n = 5–6/group). (**L**) *mPFC brain*-*derived neurotrophic factor* (*BDNF*) *expression in the F1 generation*. Representative coronal plate (AP 2.70 mm) and region of interest (ROI-gray rectangular) of mPFC. *i*-*iv*: BDNF expression (scale bar = 500 μm). (**M**,**N**) Standard animals, particularly females, born to social mothers expressed more BDNF mRNA and protein in the mPFC. Circles in the bar graphs represent individual animals. Asterisks indicate significant differences: *p ≤ 0.05; *One*-*way* and *Repeated*-*measures* ANOVA. Error bars show ± SEM.

*Neuronal density*: The regions of interest (ROI) for gray value index (GVI)^14^ in the F0 generation accompanied by a summary of housing-related changes in cortical neuronal density in F0 rats (n = 7/group) is shown in Fig. 4A,B. The GVIs in both (left and right) regions of interest in animals raised in the social housing condition were higher compared to standard housing groups. There was a significant effect of Group (F_3,26_ = 23.69, p ≤ 0.01; *Repeated*-*measures* ANOVA) but not Hemisphere (p ≥ 0.068). Also, socially-housed females displayed higher cortical cell density compared to social males (0.416 ± 0.008 vs. 0.385 ± 0.009; p ≤ 0.03, *Post*-*hoc*) indicating that social interactions during development have more impact on cortical cell density in females than males. *Cortical thickness*: A summary of the procedure for cortical thickness^14^ in F0 rats is shown in Fig. 4C. Cortical thickness was measured in the medial, central, lateral, and ventrolateral portions in both hemispheres (n = 7/group). No effect of Hemisphere was found (p ≥ 0.079) but a significant effect of Group (standard-two levels vs. social-two levels; 1.525 ± 0.08 mm vs. 1.851 ± 0.09 mm; F_3,24_ = 16.33, p ≤ 0.02; *Repeated*-*measures* ANOVA) and Region (four levels; F_3,24_ = 26.18, p ≤ 0.01; *Repeated*-*measures* ANOVA). No effect of Litter, and interaction was found between factors. Social animals had significantly thicker cortices when compared with standard groups (all p ≤ 0.05, *Post*-*hoc*, Fig. 4D). Thus, social experience during development may particularly improve cortical cytoarchitectonics in female rats.

### Maternal social experience in F1 females increased PFC dendritic complexity and spine density

A family history of social experience significantly affected neuronal morphology in offspring via the maternal lineage (Fig. 4E–K). *Dendritic branching*: Fig. 4F illustrates changes in dendritic branching in F1 rats (n = 5–6/group) raised in standard housing. *One*-*way* ANOVA indicated an effect of Group (Apical: F_3,19_ = 14.18, p ≤ 0.03; Basilar: F_3,19_ = 18.74, p ≤ 0.02) and Sex (Apical: F_1,19_ = 12.05, p ≤ 0.03; Basilar: F_1,19_ = 9.26, p ≤ 0.04). Males and females whose mothers were raised in social housing displayed increased apical branching when compared to other groups (all p ≤ 0.05, *Post*-*hoc*). However, this difference was significant only for females (n = 6) which showed more basilar dendritic branching than other groups (33.9 ± 1.6 vs. 29.78 ± 1.7, 27.66 ± 1.7 and 25.91 ± 1.6; all p ≤ 0.05, *Post*-*hoc*, Fig. 4G). *Dendritic length*: Again, only females (n = 6) who were born to social mothers showed longer apical (53.19 ± 0.42 µ vs. 51.93 ± 0.36 µ, 51.23 ± 0.47 µ and 51.39 ± 0.37 µ; all p ≤ 0.05, *Post*-*hoc*) and basilar (56.78 ± 1.1 µ vs. 54.11 ± 1 µ, 51.83 ± 1 µ and 52.36 ± 1 µ; all p ≤ 0.05, *Post*-*hoc*) dendrites than other groups causing a significant effect of Sex (Apical: F_1,19_ = 7.11, p ≤ 0.03; *One*-*way* ANOVA), and an effect of Group (Basilar: F_3,19_ = 19.41, p ≤ 0.02; *One*-*way* ANOVA, Fig. 4H,I). *Spine density*: A significant effect of Group (F_3,19_ = 19.02, p ≤ 0.03; *One*-*way* ANOVA) was found in terms of apical spine density indicating that females from social mothers displayed greater spine density than other groups including their male counterparts (8.39 ± 0.32 vs. 7.04 ± 0.33, 6.74 ± 0.36 and 6.83 ± 0.36; all p ≤ 0.05, *Post*-*hoc*, Fig. 4J). No difference was found between groups and sexes in basilar spine density (all p ≥ 0.05, Fig. 4K). In summary, social experience in mothers alters neuronal morphology more likely in their female F1 offspring.

### Maternal social experience in F1 females increased BDNF mRNA and protein expression in the medial prefrontal cortex (mPFC)

*BDNF mRNA expression*: A representative autoradiograph of cortical BDNF mRNA expression is shown in Fig. 4L. *In situ* hybridization revealed that BDNF mRNA in F1 animals born to either standard or social mothers was abundantly and differentially expressed in the mPFC leading to a significant effect of Group (F_3,15_ = 17.34, p ≤ 0.03; *One*-*way* ANOVA) and Sex (*F*_1,15_ = 24.08, *p* = 0.02; *One*-*way* ANOVA) suggesting that F0 mothers’ social experience had a noticeable impact on BDNF mRNA expression of F1 non-social females only (n = 5; 19.71 ± 1.32 vs. 14.43 ± 1.33, 11.88 ± 1.36 and 13.9 ± 1.34; all p ≤ 0.05, *Post*-*hoc*, Fig. 4M). No effect of Hemisphere and Litter was found (all p ≥ 0.05). *BDNF protein expression*: *One*-*way* ANOVA indicated no differences between right and left PFC in terms of BDNF protein expression (*p* = 0.69). However, BDNF protein expression in male and female rats (n = 5/group) born to social mothers was significantly increased when compared with animals whose fathers were socially reared (143.64 ± 13.46 ng/g vs. 101.60 ± 13.75 ng/g; all p ≤ 0.05, *Post*-*hoc*, following an effect of Group: *F*_3,15_ = 9.37, *p* = 0.04; Fig. 4N). No other significant effect was found. Thus, enhanced BDNF mRNA and protein expression in mPFC in F1 females provide further evidence that F0 social mothers transferred their social experience at the level of molecular changes only to their female offspring.

## Discussion

The brain is flexible to accommodate a wide range of environmental stimuli, and even responds to experiences intergenerationally transmitted through the ancestors. The present study expands these findings by showing that (1) social experience has a significant impact on neuroanatomy and behaviour particularly in females; and (2) consequences of social experience in females can be transmitted to their unexposed female descendants. Specifically, social stimulation in the F0 generation reduced HPA axis activity in both males and females, which was accompanied by increased cortical thickness and neuronal density. Behaviourally, social stimulation led to more exploratory activity in F0 females only when compared with their social male siblings and with non-socialized counterparts. However, spatial and motor functions were not affected by social experience. In the offspring, F1 non-social females born to F0 social mothers displayed behavioural, anatomical, and molecular alterations that followed those changes seen in their social mothers: non-social females showed increased dendritic complexity and higher spine density along with enhanced BDNF mRNA and protein expression in the mPFC. While both males and females showed decreased HPA axis activity, only females displayed increased locomotion and reduced anxiety-related behaviours. Our findings confirm that social experience alone generates beneficial responses in a rat model that are transmitted to the F1 generation in a sexually dimorphic manner.

The present findings demonstrate that social experiences represent an integral component of enriched environment (EE) therapeutic strategies. By isolating a single element of the EE, social stimulation, from the other constituents (e.g. cognitive, sensory and motor stimulation), we challenge the main conclusion drawn from Rosenzweig’s report^1^. Our findings indicate that both neuronal density and cortical thickness in F0 animals, particularly females, were remarkably impacted by mere social experiences. Interestingly, the cortical alterations seen in F0 rats were transmitted to F1 non-social female animals in terms of higher dendritic complexities and enhanced expression of cortical BDNF mRNA and protein. The observations in social animals and their non-social descendants support the influential impact of social experience on brain structure and function in both generations.

A number of possibilities should be considered as the main source of discrepancy between the present results and the findings reported by Rosenzweig’s laboratory^1^. First, Rosenzweig’s study used a single-sex design with only males, while the present study design monitored sex-dependent differences in response to social interactions. Second and more importantly, Rosenzweig’s work did not reveal an effect of mere social grouping, perhaps because animals were exposed to social interaction for only 30 days, albeit from postnatal day 25, whereas presently animals did experience social life for more than 90 days. This procedural contrast highlights the salience of social stimulation as a function of time, i.e., its duration in neurobehavioural plasticity. Therefore, the present findings were able to profile a long-term developmental trajectory of social experience-dependent neuroplasticity and behaviour in females that has been absent in previous studies.

From a developmental perspective, the changes observed in the present study may be explained by maternal responsiveness to offspring. Maternal care behaviours, such as licking, grooming frequency and lactation history are critical to offspring survival and development^19,20^. Therefore, results can also be potentially confounded by the quality and quantity of maternal behaviours during early development, and these characteristics may even propagate across generations^21^. Previously, studies of experiential effects on parent-offspring relationships have highlighted the elemental role of epigenetic mechanisms^22^, heritable phenotypic influences that may contribute in inducing variations in the parental brain and behaviours, and their subsequent offspring. In the absence of a systematic assessment of maternal behaviours, it is difficult to rule out these influences. How such variations in maternal behaviours in social and non-social mothers and their corresponding maternal care shape sex-dependent phenotypes still awaits further investigation.

Sex-specific morphological effects induced by social stimulation also highlight the critical function of sex steroids and other hormonal correlates involved in neuroanatomical alterations. As previously reported^23^ there are fundamental differences between males and females in dendritic organization of the mPFC. Because the mPFC contains both estrogen and progesterone receptors^24^, the sexual dimorphism here can be attributed to changes in gonadal hormones in females influenced by social experience. Moreover, the neuropeptide oxytocin and related biological pathways play a key role in the anatomical and functional response to social interaction. Through oxytocin action, maternal care may be intensified, social bonding enhanced and stress responses may be dampened, thus affecting the offspring’s behavioural phenotype^25^. Moreover, oxytocin may mediate sexual dimorphisms in regional neuroplasticity^26^. Thus, along with the anxiolytic actions of oxytocin^27^, social interaction may influence brain anatomy and facilitate novelty-seeking behaviours.

The prefrontal cortex (PFC) plays a key role in the control of social behaviour^28^, including play fighting. Social behaviour especially in adolescent rats is expressed by play fighting, which is vital to the maturation of brain and behaviour^29^. In turn, lesions particularly to the mPFC lead to alterations in social play behaviour in rats^30^. Experimental manipulations of play behaviour in rats result in neuromorphological changes in the PFC^31^, and functional integrity of this region has been shown to be essential for the expression of social behaviour, including play^32^. In addition, play and social interaction are associated with sensory stimulation and motor activity. Thus, social stimulation arguably represents a key aspect of an effective EE intervention that is able to promote brain development and lifelong neuroplasticity.

Our findings reveal that hypothalamic-pituitary-adrenal (HPA) axis response in F0 rats was remarkably affected by social stimulation. Notably, both social males and females transmitted the new neurohormonal phenotype to their F1 descendants. The HPA axis is known as a neurohormonal hallmark of emotionality in animals and humans^33^, and its function was previously shown to be regulated by genetic and epigenetic factors^20^, environmental interventions^7,34^ and ancestral influences^35^. The observation that parental social experience can be translated into diminished HPA axis activity and transmitted to subsequent generation supports the concept of developmental programming of neurohormonal responses^36^. Although not fully known yet, the mechanisms likely include epigenetic regulation such as DNA methylation and histone acetylation to alter glucocorticoid receptor expression^37^ during fetal^38^, and postnatal development^34^. Thus, a specific profile of central expression of glucocorticoid receptors^39^ and mineralocorticoid receptors^40^ in socially raised animals may result in new hormonal phenotypes and arguably attenuated stress responses. Responses to novelty in rats are potentially heritable^41^. Furthermore, spontaneous locomotion or social responses in female rats are not influenced by estrus cycle^42,43^, and the estrous cycle in female rats is closely associated with CORT response, a mechanism that may also affect novelty seeking. Although the present strategy for randomization of sample selection and group assignment might diminish confounding effects of the estrous cycle, conclusions about novelty seeking behaviours and also the HPA axis activity in the absence of estrous cycle monitoring should be drawn with caution.

The changes in brain morphology may at least in part be causally linked to BDNF expression^44^. For example, increased corticosterone circulation by gestational aversive experiences has been suggested to be negatively correlated with BDNF expression in the PFC^45^. While social stimulation reduced HPA axis activity, the present observations reveal increased BDNF mRNA expression in the PFC of F1 non-social rats born to F0 social mothers. The findings extend current knowledge by showing that maternal social experience may program BDNF expression in the subsequent generation in a sexually dimorphic manner. Growing evidence suggests that adverse maternal experiences may particularly program BDNF expression and thus alter brain development and mental and emotional health in later life^46^. In general, BDNF expression seems to represent a particularly sensitive measure of socioeconomic and maternal lifestyle^47^. Moreover, paternal social experiences influence BDNF expression and offspring behaviour via the maternal lineage^48^. Through these mechanisms and others, parental experiences are able to influence behaviour and brain of their offspring and potentially even further generations^17,49^.

The main mechanism for developmental programming by environmental interventions involves altered activity of HPA axis^7^. Hypoactivity of the HPA system linked to social stimulation in both sexes and generations may also determine the behavioural phenotype. Here, the behavioural phenotype was characterized by improved novelty-seeking exploratory behaviour and reduced anxiety-related behaviour. Accordingly, our previous observations revealed that increased HPA axis activity is accompanied by impaired novelty seeking in a complex environment^10^. However, the central hypothesis underlying the use of the CFT in the present experiments was that when a rat is placed in the arena, it may involve a strong motivational propensity to stay in the corridor (anxiolytic effect), and also induces an emotional situation through perceived threat within the open area (anxiogenic effect). Therefore, the corridor and open zones in the CFT depict a more dynamic picture of the psychological, mainly motivational correlates of exploratory behaviour than the classic open-field task (OFT). While the OFT seems an anxiogenic task by nature^50^, and open-field activity provides the primary indicator of emotionality^15^, the specific physical characteristics of the CFT appear to impose more motivational inhibitions to the animals, thus providing a measure of motivational levels of exploratory behaviours.

The central object in the CFT was employed to encourage exploration and/or to enhance novelty-seeking behaviour^51^. The F0 mothers who experienced social stimulation as well as their female F1 offspring extended the level of exploration to the central areas, thus growing the radius of exploratory activity and indicating lowered stress response^10^. These data not only confirm that the CFT is ideally suited to differentiate experience-dependent psychomotor profiles, but also replicates the literature of experience-dependent novelty-seeking exploratory behaviours in a new framework. They also confirm that females seem to be more responsive to social stimulation than males.

Experiences, such as social stimulation, may generate robust phenotypes that may even be transmitted to the offspring through physiological programming^36^ or epigenetic mechanisms^52^, which then determine stress sensitivity in the F1 generation and beyond. Early experiences in particular, including prenatal stress, malnutrition or exposure to toxicants, seem to be the most salient to result in phenotype programming in the offspring^52^. A stress-sensitive phenotype is usually linked to downregulation in hippocampal and cortical glucocorticoid receptor and neuronal densities^7^. Through these mechanisms ancestral experiences may promote the formation of new behavioural phenotypes in subsequent generations^49,53^ via the paternal or maternal lineages^54,55^. Moreover, early-gestation stress translates into adverse consequences for brain development, behaviour, and overall health^37^ and is associated with epigenetic inheritance of a stress-sensitive phenotype in second-generation offspring of the male lineage^22^ and third-generation offspring in the female lineage^53^. While most research focused on phenotype programming by an early environment, experiences in later life may also produce stress-sensitive phenotypes^56^. The present data show that the pre-conceptional social environment generates a phenotype that may be transmitted to subsequent generations in a sex-dependent manner.

## Conclusions

The classic paradigm of EE combines inanimate and social interaction although there is no consensus yet on which EE paradigm is ideal to promote brain development and behaviour^57^. Unlike the classic EE model, our paradigm isolated the impact of social stimulation to show that social experience generates new sex-dependent neurohormonal and behavioural phenotypes. In agreement with Welch and others^8^, we report in rats that mere social experience can change brain structure and behaviour. Moreover, we show that social experience-induced alterations can be transmitted to the non-exposed offspring. The data suggest that females more than males benefit from a socially stimulating environment. The present findings show that social mothers predominantly transfer the newly formed phenotype to their non-social daughters through mechanisms involving altered HPA axis response and BDNF expression. Programming of the experience- and sex-dependent phenotype is arguably generated via epigenetic regulation. Additional novelty and complexity of the environment (e.g. greater levels of sensory stimulation and physical activity) may have further promoted structural and functional improvements as reported in traditional EE models. Nevertheless, the present findings represent a stepping stone for further investigation of stress-associated psychomotor pathologies and related endocrine and epigenetic biomarkers, and their mitigation by social stimulation. Most importantly, a socially stimulating environment may moderate these pathologies not only in the afflicted individuals, but also in their intergenerationally programmed descendants.
